# Exercise intensity agreement, need satisfaction, and exercise behavior: A sex‐moderated mediation model

**DOI:** 10.1002/ejsc.12173

**Published:** 2024-09-16

**Authors:** D. S. Teixeira, A. J. Andrade, J. Faria, P. Marques, V. Bastos, F. Rodrigues, A. M. Sousa, H. V. Pereira

**Affiliations:** ^1^ Faculty of Physical Education and Sport Lusófona University of Humanities and Technologies Lisbon Portugal; ^2^ Research Center in Sport, Physical Education, and Exercise and Health (CIDEFES) Lisbon Portugal; ^3^ ESECS Polytechnique of Leiria Leiria Portugal; ^4^ Centro de Investigação em Desporto Saúde e Desenvolvimento Humano (CIDESD) Covilhã Portugal

**Keywords:** adherence, exercise, hedonism, motivation, professionals

## Abstract

Several theoretical frameworks have been used to understand exercise adherence. Basic psychological needs (BPN), grounded on self‐determination theory, have received large attention for this purpose. More recently, the hedonic theory and the assumptions related to the exercise characteristics (e.g., intensity) that can bolster positive affective responses have been revitalized. This study aimed to explore the associations between the agreement of current exercise intensity and the one individually preferred, BPN satisfaction/frustration, enjoyment, the intention to continue exercise, and exercise frequency. Additionally, an exploration of the direct and indirect effects while testing sex as a moderator was performed. A sample of 369 exercisers (*M*_age_ = 43.6, standard deviation = 12.96; 214 females) enrolled in 26 health clubs participated in this study voluntarily. Data were received in a first‐approach descriptive and correlational analyses. Next, a moderated mediation was performed using model 15 (PROCESS v.4.2). As a result, agreement in preference for exercise intensity was positively associated with enjoyment (*r* = 0.35), intention (*r* = 0.43), and all needs satisfaction (ranging from *r* = 0.12 to *r* = 0.45) and negatively associated with all needs frustration (ranging from *r* = −0.15 to *r* = −0.31). In the moderated mediation analysis, the same pattern of results emerged in direct effects. Indirect effects were significant for autonomy in the enjoyment and frequency models. Findings suggest that promoting an individually adjusted training intensity may foster BPN satisfaction. It appears to be present an independent (of needs) and positive association with exercise enjoyment and intention to continue exercising.

## Highlights

- An agreement between exercise intensity and the one individually preferred can be seen as a need‐supportive tool
- Exercise intensity agreement presents an independent effect on exercise enjoyment and intention
- The way autonomy promotes enjoyment may be different between sexes, a relation that seems to favor men
- Females appear to be more dependent on preference agreement to support enjoyment and intention, while in men, this relation appears to be valid only for intention

## 1 INTRODUCTION

Promoting physical exercise adherence is challenging. Given the complexity of human behavior, several factors can influence one's decision to be continuously involved in such an effort. Among the several approaches that can be developed to help face this issue, motivation has been the focus of extensive research (Ntoumanis et al., 2018). Of the several theories and frameworks with differentiated outcomes and approaches, self‐determination theory (SDT; Deci & Ryan, 1985) has been one of the most explored and suggested for this purpose (Rhodes et al., 2019; Ryan et al., 2021, 2022).

Within the tenets of SDT, basic psychological needs (BPN) emerge as three universal psychological nutrients (autonomy: being able to choose the behavior or to be in control of it; competence: one's ability to succeed in challenging tasks and attain desired outcomes; and relatedness: development of personal and meaningful connections based on trust and respect) which, when satisfied, are considered essential for personal growth and well‐being, and for behavioral stability and positive psychological outcomes (Ryan & Deci, 2017). On the contrary, BPN frustration acts oppositely and is proposed to be detrimental to one's health, well‐being, and motivational quality (Ryan & Deci, 2017). Additionally, BPN satisfaction is postulated to be a facilitator of behavior internalization and integration, allowing individuals to regulate their efforts more autonomously (i.e., behavior performed out of personal interest or inherent value) (Ryan & Deci, 2017), which is proposed to sustain intrinsic motivation and exercise adherence (Ntoumanis et al., 2018, 2021; Ryan et al., 2021, 2022).

Promoting the development of BPN in an exercise context is difficult. The articulation between customary professional practice (i.e., related to exercise prescription and supervision) and motivational considerations and strategies often relegates the latter to the second plan, with professionals being more at ease with current and known exercise variables and less with motivational aspects related to exercise adherence. However, emerging theoretical suggestions highlight the potential of adjusting the prescription and supervision processes aiming to reach individual exercise preferences to ease the introduction of motivational strategies and assumptions that can lead to, for example, the development of BPN. Exercise intensity manipulation is one of those proposals, where the approximation between current exercise intensity and the one individually preferred is proposed as being need supportive (Teixeira, Rodrigues, et al., 2021; Rodrigues et al., 2023). As such, much potential for exercise motivation and behavior is promised if professionals can operationally support BPN development in their regular professional practices by adjusting standard prescribing variables.

### 1.1 Contextual promotion of BPN—The potential role of intensity preference agreement

In a supervised exercise context, exercise evaluation and prescription are two core aspects of the professional intervention. Notwithstanding, professionals must be aware that for sustained exercise practice, they must find a compromise between what they judge to be physiologically best for their clients (as for their goals and expectations), while at the same time exploring strategies to support the behavior across time (American College of Sport Science [ACSM], 2021; Teixeira, Rodrigues, et al., 2021). This is challenging for different reasons. For once, professionals must manage several exercise training variables (e.g., intensity, cadence/speed, and volume), as for the exercise technique and performance, which is highly time and focus demanding in a supervised process. Additionally, current exercise promotion strategies and recommendations present several frameworks and techniques (e.g., for a brief review, see chapter 12 of the ACSM (2021) guidelines) that professionals must be able to select for each client and at each moment. Because these environments and interactions are highly dynamic, the implementation of these behavioral guidelines is not straightforward. Additionally, for some exercise professionals, the gap between knowing some motivational theories/behavior change techniques and being able to apply them *operationally* may be a considerable shortcoming for their successful implementation (Hancox et al., 2017). For that matter, a considerable amount of work is yet to be done concerning effectively operationalizing the several recommendations emerging from the motivational and behavioral frameworks in the most common exercise contexts from the professional operational standpoint.

One key variable in exercise prescription is intensity. All professionals need to know how to select and manipulate this variable for all exercise modes and any type of individual goals and characteristics. It is present across all stages of an exercise session and planning and can be monitored and assessed through several methods and instruments such as heart rate monitor, perceived effort, or repetitions in reserve (ACSM, 2021; Schoenfeld, 2021).

Several approaches have been performed throughout the last decades to understand the potential role of intensity on exercise adherence. Some straightforward indications suggest that an individually tailored exercise intensity tends to support exercise behavior and motivation, for example, through a hedonic approach (i.e., pleasure/displeasure in a given activity) (Ekkekakis et al., 2011; Stevens et al., 2020; Teixeira et al., 2022). As reported extensively, higher exercise intensities tend to promote in most individuals unpleasant sensations during exercise, while lower intensities tend to promote the opposite, at least until the ventilatory threshold (Ekkekakis et al., 2011), high exercise loads (>75% of repetition maximum) (Andrade et al., 2022), or vigorous stretching intensities (Henriques et al., 2023).

Aiming to understand better how intensity could be individually assessed, Ekkekakis et al. (2005) developed an instrument called the Preference for and Tolerance of Exercise Questionnaire (PRETIE‐Q), which intends to evaluate how each individual prefers and tolerates the exposure to exercise intensity. These and other subsequent authors claimed that these two constructs could be used, among other variables, to help professionals select exercise intensity when targeting improved feelings (e.g., affective response) during exercise that would be conducive to adjusted exercise motivation and adherence (Ekkekakis et al., 2005; Teixeira, Ekkekakis, et al., 2021; Teixeira et al., 2023). Additionally, Teixeira, Ekkekakis, et al. (2021) have extended the use of this instrument with two additional items and proposed that the level of agreement between the exercise intensity and the one preferred and tolerated by an individual could be helpful in the understanding and improvement of motivation and exercise behavior. Several works have been developed grounded on this assumption (for a review, see Santos & Teixeira, 2023), and evidence has emerged suggesting that when targeting the agreement of these two constructs, improved indicators of exercise behavior (e.g., frequency, adherence, intention, and habit) (Faria et al., 2021; Marques et al., 2022; Teixeira et al., 2022), subjective vitality (Faria et al., 2021; Teixeira, Ekkekakis, et al., 2021), and enjoyment (Teixeira, Rodrigues, et al., 2021; Teixeira et al., 2022) were present.

Regarding motivation as presented by the SDT, only two studies were found to test this framework and preference/tolerance constructs. First, in Teixeira, Rodrigues, et al. (2021), preference and tolerance were tested as dispositional constructs on exercise enjoyment using BPN satisfaction and frustration as mediators. They found some effects of preference and tolerance on BPN satisfaction (positive associations) and frustration (negative associations) and proposed the existence of direct and indirect effects that could shape several motivational constructs. However, they hypothesized that *“the intensity‐traits agreement with the exercise training should be supportive of needs satisfaction”* (p. 3) without effectively testing the participant's level of agreement. Moreover, their exploration used a composite approach to BPN satisfaction and frustration testing, which did not allow for an understanding of how each need could be interpreted in the studied relationships. Posteriorly, Rodrigues et al. (2023), grounded on some suggestions of Teixeira, Rodrigues, et al. (2021), tested the relationships between preference and tolerance on the BPN of competence (satisfaction and frustration) and behavioral intention. They found that preference was a positive predictor of competence satisfaction and intention, but no evidence emerged for the role of tolerance on these variables. Once again, no formal testing of the level of agreement was made, leaving the aforementioned hypothesis untested.

Reasons to suspect that how exercise intensity is aligned with individual preferences may promote BPN satisfaction are diverse. Teixeira, Rodrigues, et al. (2021) indicated that the exerciser may perceive a guided/supervised self‐selected exercise intensity as autonomy‐supportive, given that it allows the individual to (albeit partially) choose one's behavior. Giving a sense of control and choice during exercise has, in fact, been seen as one of many approaches to support autonomy in exercise settings operationally (and also regarding intensity choice; Andrade et al., 2024; Vazou & Ekkekakis, 2009) and is generally a recommended approach for that endeavor (Kwasnicka et al., 2016; Ryan & Deci, 2017). Additionally, exercise intensity is closely related to how an exercise can be performed. For example, high exercise intensity in a training session may hinder proper technique, leading to pain/discomfort, an unadjusted exercise experience, and ultimately act as a deterrent for individual goal achievement, which may, in general, compromise competence perceptions (Rodrigues et al., 2023). As posited by Teixeira, Rodrigues, et al. (2021, p. 3), *“activities performed at a self‐selected intensity may aid developing competence and mastery that is adjusted to the actual level of ability, capabilities and interest”*, which seems like a valid hypothesis, but yet to be tested. Finally, the definition of an exercise intensity that is aligned with individual preferences may add to the establishment of meaningful interactions between the exerciser and the professional if the exerciser perceives this intensity prescription or the possibility of a guided self‐selection/regulation as a way of expressing concern and giving emotional support to individual needs and difficulties. This would facilitate the development of trust and respect, both core aspects of relatedness satisfaction (Ryan & Deci, 2017; Teixeira, Rodrigues, et al. (2021)). Opposingly, imposing exercise intensity may not only hinder needs satisfaction but also actively frustrate them (Teixeira et al., 2018), something that can easily occur by selecting an exercise intensity beyond what the individual wants or feels prepared or interested in doing in a given moment (i.e., absence of intensity preference agreement). Autonomy, for example, may be hindered by limiting one's will in detriment of an excessive focus on results (e.g., no pain, no gain mentality) (Pereira et al., 2022); competence may suffer the same effect given an excessive exercise intensity demand, which may difficult the activity realization (e.g., premature termination of the exercise/session), thus affecting a sense of achievement and contentment experienced in the activity (Rodrigues et al., 2023); as for relatedness, when no effective and meaningful connection is present within the exerciser–professional interaction, the unadjusted imposed intensity may be seem as an uninterested, undifferentiated, or “cold” approach, and thus disregarding individual needs (McEwan et al., 2021; Teixeira & Palmeira, 2016).

### 1.2 Present study

SDT proposes that a need‐supportive environment and respective strategies should lead to BPN fulfillment, improved motivation quality (i.e., increased autonomous motivation), and impact exercise behavior (e.g., through exercise persistence) (Rodrigues et al., 2018, 2020; Ryan & Deci, 2017). Additionally, the BPN development is proposed to be promoted using multiple co‐acting techniques (Gillison et al., 2019). However, despite some efforts, very few works have addressed and tested how to operationalize a need‐supportive environment in exercise settings (e.g., Hancox et al., 2017; Rodrigues, Teixeira, et al., 2019). For this matter, the present work intended to explore the relationship between the agreement of current exercise intensity and the one individually preferred and the respective associations with BPN satisfaction/frustration, enjoyment, intention, and exercise frequency. Additionally, sex will be tested for effects on the preference agreement and all outcome variables, as for all mediator's relations with all outcome variables. It is hypothesized that the exercisers will perceive intensity preference agreement as a facilitator of competence, relatedness, but particularly, autonomy satisfaction, and that this agreement will be negatively associated with needs frustration (Teixeira, Rodrigues, et al., 2021, 2022). Preference agreement is also hypothesized to be positively associated with enjoyment and intention to continue exercising (Marques et al., 2022; Teixeira, Rodrigues, et al., 2021, 2022), two known predictors of exercise behavior, and also with exercise frequency (Teixeira et al., 2022). Finally, BPNs are expected to act as mediators in the preference agreement and (i) enjoyment, (ii) intention to exercise in the future, and (iii) exercise frequency outcomes, given their well‐known associations with these variables (e.g., Rodrigues et al., 2020; Teixeira et al., 2018), and the associations expected in the first hypothesis. No role for sex is expected between the preference agreement and the BPN's relation, given previous theoretical indications, and construct invariance and latent means absence of differences (Ekkekakis et al., 2008; Santos & Teixeira, 2023). However, it is expected that in all paths leading to the outcome's variables, sex could moderate some effects. For example, men and women tend to differ in the way exercise‐related pain and fatigue is experienced (Ansdell et al., 2020; Rascon et al., 2020), which may justify distinct exercise intensity selection when given that possibility; moreover, men may tend to prefer higher intensity activities (Cobbold, 2018; Reading & LaRose, 2022). These perceptions (i.e., inter‐sex experienced exercise intensity) are expected to influence exercise enjoyment, intentions to be (or continue to be) physically active, and the amount of exercise practiced (Astorino & Sheard, 2019; Teixeira et al., 2022). Moreover, individual exercise prescription tailoring, something preference agreement may help achieve, is suggested to be dependent of specific contextual influences, where precursors of autonomy support, as is the case of BPN, may be vital prerequisites for certain groups of exercisers (as is the case of sex) (Josefsson et al., 2018; Mullen & Whaley, 2010; Rodrigues et al., 2022).

## 2 METHOD

### 2.1 Participants and procedures

We followed Fritz and MacKinnon (2007), Hayes (2018), and Ma and Zeng (2014) recommendations for sample size definition. First, we checked simulations of multiple mediation analysis where 1‐*β* = 0.80 and *f* ^2^ = 0.15 were tested and found that a sample size >200 was recommended. Then, we tested the sample size needed in a moderation model with seven interactions (per model) using G*Power (Faul et al., 2009), using an *f* ^2^ = 0.15, *α* = 0.05, a 1‐*β* = 0.80, indicating the need for a sample with 103 individuals. Given that no clear indication exists on how to calculate the adequate sample size for a moderated mediation analysis with the present characteristics, we choose to acquire a sample that would considerably surpass the aforementioned sample size calculations. Additionally, we also used a bootstrapping resampling procedure (see method) to protect from type I error rates.

A sample of 369 health club exercisers (*M*_age_ = 43.6, standard deviation (SD) = 12.96; 214 females) enrolled in 26 health clubs representing all districts in Portugal participated voluntarily in this study. Participants were involved in distinct activities (e.g., personal training; group classes), had an average experience in this setting of practice of 11.05 years (SD = 9.29), and attended the club 3.96 times/week on average (SD = 1.20). They classified their health club experience on a five‐point scale (1—“Very bad”; 5—“Very good”) as being good (*M* = 4.4; SD = 0.67), and 64.6% (*n* = 168) of the exercisers were training with and exercise plan and/or supervision (109 individuals' response on this variable were lost due to technical issues during the collection). Inclusion criteria defined that participants should be >18 years old and apparently healthy (no limitations to exercise participation).

The present study data consist of part of the baseline data of a larger and ongoing longitudinal research project related to the quality of the subjective exercise experience in health clubs, where prior ethical approval from the first author institution was obtained (omitted for review purposes). The technical directors of the health clubs were contacted to provide permission for data collection. A letter of explanation with the study aim, inclusion criteria, expected participation, data confidentiality, possibility to cease participation at any moment, and the email contact of the study responsible was present at the beginning of the questionnaires. After that, written consent was asked and required for study participation. Upon written permission, the battery of questionnaires was made available in the reception of the clubs and exercisers invited to participate. All the procedures were developed according to the Helsinki Declaration and its later amendments. The total time expected to fulfill the questionnaires was ∼15 min.

### 2.2 Measures

*Individual intensity preference*. The preference agreement item from the Preference for and Tolerance of the Intensity of Exercise Questionnaire Portuguese version (PRETIE‐Q‐PT; Teixeira, Ekkekakis, et al., 2021) was used to assess the degree of agreement between the individual preference and the current exercise intensity *(“The intensity of my training is in accordance with my preference”*). This item on PRETIE‐Q‐PT presented a dichotomous possibility of response (0—not in agreement; one—in agreement), but other studies (e.g., Santos & Teixeira, 2023) have suggested a 5‐point Likert scale ranging from 1 *(“Totally disagree”*) to 5 (*“Totally agree”*) to ensure a more detailed understanding of the intended agreement understanding. In this study, we followed their recommendations.

*Basic psychological needs*. The BPN Satisfaction and Frustration Scale in Exercise Portuguese version (BPNSFS‐E; Rodrigues, Hair, et al., 2019) assessed needs satisfaction and frustration. Of the 24 items, 12 evaluate BPN satisfaction (autonomy, e.g., *“I have a feeling of freedom and choice in the things I make”*; competence, e.g., *“I feel confident that I can do things right”*; relatedness, e.g., “*I feel that the people I care for, also care for me”*), and the other 12 BPN frustration (autonomy, e.g., *“I feel the majority of the things I do out of obligation”*; competence, e.g., *“I feel insecure of my abilities”*; relatedness, e.g., *“I feel excluded from the group I want to belong”*). Answers were given using a 5‐point Likert scale ranging from 1 *(“Totally disagree”*) to 5 *(“Totally agree”*). The current instrument is aligned with SDT recommendations and has provided previous validity for the intended purposes (Ryan & Deci, 2017; Vansteenkiste et al., 2020).

*Exercise enjoyment*. Perceptions of the degree of exercise enjoyment were obtained using an instrument composed of eight items (e.g., *“It is fun”*) answered on a 7‐point Likert scale ranging from 1 (*“Totally disagree”*) to 7 (*“Totally agree”*), following similar studies in the same context (e.g., Teixeira et al., 2023).

*Intention to continue exercising*. Behavioral intentions were obtained through three questions (e.g., *“I will continue to practice physical exercise in the next* 6 months *as I recently practiced or in a very similar way”*) based on Ajzen's (2006) recommendations and previously used in similar contexts (Rodrigues et al., 2020). Responses were given using a 7‐point bipolar scale ranging from 1 (*“Absolutely not”*) to 7 (*“Absolutely yes”*).

*Exercise frequency*. Exercise weekly frequency was obtained objectively through the health club's digital records obtained each time the exerciser crossed the turnstile. The previous three months' records were used to define the average weekly exercise frequency for baseline purposes.

### 2.3 Statistical analysis

Descriptive statistics, reliability, and bivariate correlations were developed for all variables. Data were screened for missing values, and in cases with more than 5% of absent data (1.55%), participants were removed before the analysis. In cases where absent data were <5%, cases were analyzed for the possibility of data imputation procedures (Allison, 2000). All analyses were performed using IBM SPSS Statistics v. 26.0 for Windows (IBM Co., United States), and the level of statistical significance was set at *p* < 0.05.

For the moderated mediation analysis, PROCESS v.4.2 macro for SPSS v.26.0 and model 15 were used, as recommended by Hayes (2018). This model assumes the existence of an independent variable (preference agreement), parallel mediators (six: autonomy, competence, and relatedness satisfaction; autonomy, competence, and relatedness frustration), a dependent variable (separately: enjoyment [model A], intention [model B], frequency [model C]), and a moderator (sex; tested in the independent > dependent variable path, and mediators > dependent variable paths). A bootstrap with 5000 samples was used for CI 95% intervals estimation, and significant effects were considered if CI did not encompass zero.

## 3 RESULTS

After initial screening for analysis assumptions, no issues were detected. The descriptive, reliability, and correlational results are presented in Table 1. Preference agreement, all BPN (satisfaction), enjoyment, and intention to continue exercise, depicted high mean scores (all above midpoint scores); exercise frequency showed a frequency of 3.96 times/per week. All BPN (frustration) depicted low mean scores (all below midpoint scores).

**TABLE 1:** Descriptive, reliability, and correlational analysis of the studied variables.

	*α*	*M*	SD	1	2	3	4	5	6	7	8	9
1. Preference agreement	‐	4.44	0.79	‐								
2. Autonomy satisfaction	0.739	4.27	0.62	0.445**	‐							
3. Competence satisfaction	0.797	4.42	0.57	0.293**	0.626**	‐						
4. Relatedness satisfaction	0.833	4.21	0.80	0.123*	0.407**	0.418**	‐					
5. Autonomy frustration	0.815	1.74	0.80	−0.314**	−0.500**	−0.433**	−0.200**	‐				
6. Competence frustration	0.735	1.74	0.74	−0.230**	−0.430**	−0.572**	−0.174**	0.583**	‐			
7. Relatedness frustration	0.738	1.44	0.59	−0.147**	−0.249**	−0.421**	−0.359**	0.502**	0.553**	‐		
8. Enjoyment	0.942	6.10	0.92	0.353**	0.593**	0.500**	0.314**	−0.468**	−0.341**	−0.292**	‐	
9. Intention to continue	0.943	6.40	0.96	0.426**	0.294**	0.158**	0.086	−0.297	−0.141**	−0.105*	0.216**	‐
10. Exercise frequency	‐	3.96	1.20	0.063	0.135**	0.045	0.012	−0.168**	−0.088	−0.023	0.197**	0.135**

Abbreviations: *α*, Cronbach's alpha; *M*, mean; SD, standard deviation.

*p < 0.05; **p < 0.01.

As for the bivariate correlation results, positive associations were found between preference agreement and autonomy (*r* = 0.45, *p* < 0.01), competence (*r* = 0.29, *p* < 0.01), relatedness (*r* = 0.12, *p* < 0.05), enjoyment (*r* = 0.35, *p* < 0.01), and intention (*r* = 0.43, *p* < 0.01); negative associations were found with all autonomy frustration (*r* = −0.32, *p* < 0.01), competence frustration (*r* = −0.23, *p* < 0.01), and relatedness frustration (*r* = −0.15, *p* < 0.01). Autonomy satisfaction (*r* = 0.14, *p* < 0.01), enjoyment (*r* = 0.20, *p* < 0.01), and intention (*r* = 0.14, *p* < 0.01) were positively associated with exercise frequency; autonomy frustration was negatively associated with exercise frequency (*r* = −0.17, *p* < 0.01).

**FIGURE 1:**
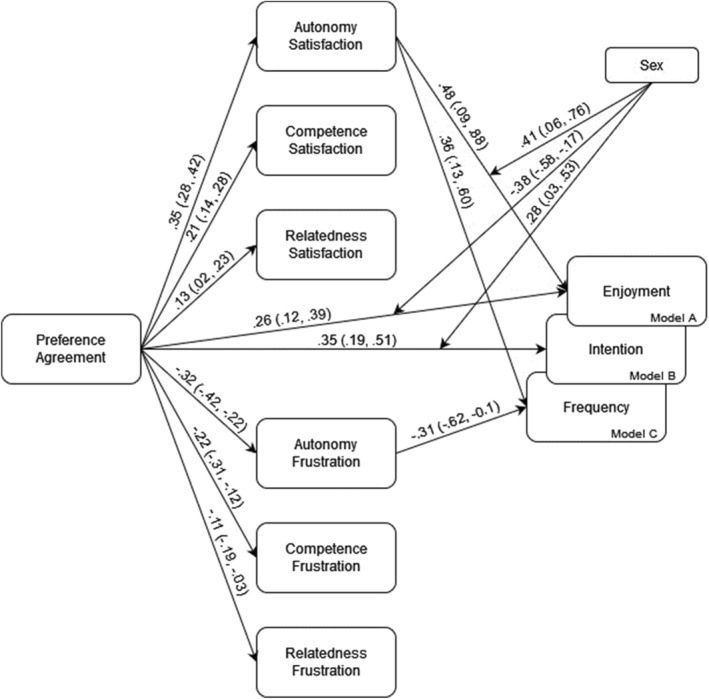
Parallel mediation models for preference agreement, BPN satisfaction and frustration and enjoyment (A), intention (B), and frequency (C). *Note.* Existing lines represent significant paths.

The moderated mediation analysis results are depicted in Figure 1 (direct paths represent the dichotomous option female) and Table 2. Positive associations were found between preference agreement and all BPN satisfaction (autonomy: *β* = 0.35, [0.28, 0.42]; competence: *β* = 0.21, [0.14, 0.28]; and relatedness: *β* = 0.13, [0.02, 0.23]); negative associations were found with all BPN frustration (autonomy frustration: *β* = −0.32, [−0.42, −0.22]; competence frustration: *β* = −0.22, [−0.31, −0.12]; and relatedness frustration: *β* = −0.11, [−0.19, −0.03]). In the three models tested, significant direct effects were found in model A (preference agreement—enjoyment; *β* = 0.26, [0.12, 0.39]) and model B (preference agreement—intention; *β* = 0.35, [0.19, 0.51]). Finally, autonomy satisfaction was positively associated with enjoyment (*β* = 0.48, [0.09, 0.88]) and exercise frequency (*β* = 0.36, [0.13, 0.60]); autonomy frustration was negatively associated with exercise frequency (*β* = −0.31, [−0.62, −0.01]). All of these represented significant indirect effects through the respective mediator.

**TABLE 2:** Direct and indirect regression scores, interactions, and conditional effects of the proposed models.

	Enjoyment (model A)				Intention (model B)				Frequency (model C)			
	Beta	SE	*t*	*P*	Beta	SE	*t*	*p*	Beta	SE	*t*	*p*
Direct effect (IV > DV)												
Preference agreement	0.26	0.07	3.8	<0.001	0.35	0.08	4.31	<0.001	−0.06	0.11	−0.55	0.58
Indirect effects (IV > M)												
Autonomy satisfaction	0.35	0.04	9.42	<0.001	0.35	0.04	9.42	<0.001	0.35	0.04	9.42	<0.001
Competence satisfaction	0.21	0.04	5.77	<0.001	0.21	0.04	5.77	<0.001	0.21	0.04	5.77	<0.001
Relatedness satisfaction	0.13	0.05	2.47	0.01	0.13	0.05	2.47	0.01	0.13	0.05	2.47	0.01
Autonomy frustration	−0.32	0.05	−6.23	<0.001	−0.32	0.05	−6.23	<0.001	−0.32	0.05	−6.23	<0.001
Competence frustration	−0.22	0.05	−4.47	<0.001	−0.22	0.05	−4.47	<0.001	−0.22	0.05	−4.47	<0.001
Relatedness frustration	−0.11	0.04	−2.83	0.005	−0.11	0.04	−2.83	0.005	−0.11	0.04	−2.83	0.005

	Beta	*t*	LLCI	ULCI	Beta	*t*	LLCI	ULCI	Beta	*t*	LLCI	ULCI
Indirect effects (M > DV)												
Autonomy satisfaction	0.36	3.08	0.132	0.598	0.233	1.64	−0.047	0.514	0.48	0.20	0.088	0.88
Competence satisfaction	0.24	1.93	−0.005	0.491	−0.168	−1.11	−0.466	0.130	−0.35	0.21	−0.077	0.072
Relatedness satisfaction	0.05	0.79	−0.078	0.183	0.095	1.18	−0.062	0.252	0.13	0.11	−0.09	0.355
Autonomy frustration	−0.16	−1.71	−0.34	0.024	−0.206	−1.85	−0.424	0.013	−0.31	0.16	−0.615	−0.001
Competence frustration	0.08	0.82	−0.11	0.269	−0.149	−1.28	−0.377	0.079	0.01	0.16	−0.314	0.33
Relatedness frustration	−0.08	−0.66	−0.323	0.16	0.155	1.047	−0.136	0.445	0.05	0.21	−0.361	0.46
Sex	−0.39	−0.39	−2.389	1.6	−1.622	−1.33	−4.024	0.78	1.66	1.73	−1.73	5.06
Interaction 1 (preference agreem. x sex)	−0.38	−3.58	−0.585	−0.17	0.281	2.22	0.032	0.530	0.100	0.57	−0.251	0.454
Interaction 2 (autonomy sat. x sex)	0.41	2.29	0.058	0.761	−0.267	−1.24	−0.690	0.156	−0.510	−1.69	−1.111	0.083
Interaction 3 (competence sat. x sex)	0.15	0.76	−0.232	0.525	0.36	1.56	−0.095	0.816	0.360	1.11	−0.279	1.007
Interaction 4 (relatedness sat. x sex)	−0.07	−0.62	−0.300	0.156	−0.186	−1.33	−0.460	0.088	−0.400	−2.02	−0.786	−0.012
Interaction 5 (autonomy frust. x sex)	−0.07	−0.58	−0.326	0.177	−0.012	−0.08	−0.314	0.291	0.030	0.14	−0.396	0.458
Interaction 6 (competence frust. x sex)	−0.03	−0.22	−0.321	0.256	0.467	2.65	0.120	0.814	0.010	0.01	−0.487	0.494
Interaction 7 (relatedness frust. x sex)	−0.02	−0.1	−0.353	0.317	−0.136	−0.66	−0.539	0.267	0.100	0.33	−0.473	0.666

	Beta	SE	LLCI	ULCI	Beta	SE	LLCI	ULCI	Beta	SE	LLCI	ULCI
Conditional direct effect (IV > DV)												
Preference agreement (sex = 0)	0.26	0.07	0.124	0.389	0.35	0.08	0.190	0.509	−0.06	0.11	−0.289	0.161
Preference agreement (sex = 1)	−0.12	0.08	−0.281	0.038	0.63	0.10	0.438	0.822	0.04	0.14	−0.233	0.309
Conditional indirect effects (IV > M > DV)												
Autonomy satisfaction (sex = 0)	0.13	0.05	0.032	0.234	0.082	0.05	−0.019	0.200	0.170	0.08	0.025	0.327
Autonomy satisfaction (sex = 1)	0.27	0.07	0.134	0.424	−0.012	0.06	−0.122	0.110	−0.010	0.08	−0.159	0.147
Competence satisfaction (sex = 0)	0.05	0.03	−0.009	0.119	−0.035	0.05	−0.142	0.046	−0.070	0.05	−0.180	0.010
Competence satisfaction (sex = 1)	0.08	0.04	0.004	0.171	0.040	0.04	−0.043	0.112	0.010	0.05	−0.100	0.100
Relatedness satisfaction (sex = 0)	0.01	0.01	−0.011	0.029	0.012	0.01	−0.007	0.038	0.020	0.02	−0.015	0.057
Relatedness satisfaction (sex = 1)	−0.01	0.01	−0.035	0.025	−0.011	0.02	−0.050	0.022	−0.030	0.03	−0.097	0.005
Autonomy frustration (sex = 0)	0.05	0.04	−0.011	0.129	0.067	0.04	0.001	0.146	0.100	0.05	0.009	0.197
Autonomy frustration (sex = 1)	0.07	0.03	0.014	0.147	0.069	0.05	−0.020	0.181	0.090	0.05	−0.002	0.187
Competence frustration (sex = 0)	−0.02	0.02	−0.066	0.025	0.032	0.03	−0.025	0.104	−0.002	0.03	−0.064	0.064
Competence frustration (sex = 1)	−0.01	0.03	−0.067	0.041	−0.070	0.04	−0.150	0.001	−0.003	0.04	−0.082	0.083
Relatedness frustration (sex = 0)	0.01	0.02	−0.016	0.047	0.017	0.02	−0.063	0.027	−0.010	0.03	0.053	0.054
Relatedness frustration (sex = 1)	0.01	0.02	−0.023	0.051	−0.002	0.02	−0.037	0.025	−0.020	0.02	−0.059	0.027

*Note*: 0 = female; 1 = male.

As for the moderation effects and interactions, sex was found to have significant interactions with preference agreement (*β* = −0.38, [−0.59, −0.17]) and autonomy satisfaction (*β* = 0.41, [0.06, 0.76]) in model A and with preference agreement (*β* = 0.28, [0.03, 0.53]) in model B. As for conditional effects in model A, significant effects were found for females in the preference agreement and enjoyment relation (*β* = 0.26, [0.12, 0.39]) and in the autonomy satisfaction—enjoyment relation for both sexes (male: *β* = 0.27, [0.13, 0.42]; female: *β* = 0.13, [0.03, 0.23]); for model B, significant conditional effects were found for both sexes in the preference agreement and intention relation (male: *β* = 0.63, [0.44, 0.82]; female: *β* = 0.35, [0.19, 0.51]). Other smaller but significant interactions are presented in Table 2. Participant's descriptive statistics and sex differences are presented in Supporting Information S1.

## 4 DISCUSSION

The present study sought to explore if the individual preference for exercise intensity and its alignment with current practice exercise intensity would indicate exercise BPN fulfillment, enjoyment, intention, and frequency. For this, direct and indirect effects were tested to understand the potential role of the theoretically proposed relationships. Additionally, sex was tested as a moderator in all associations with the independent variables.

This study's results suggest that the alignment of current training exercise intensity and the one individually preferred is positively associated with exercise BPN satisfaction and negatively associated with BPN frustration, thus supporting our first hypothesis. Also, preference agreement was positively associated with exercise enjoyment and intention to continue exercise but not with exercise frequency, which partially supported our second hypothesis. Finally, autonomy satisfaction partially mediated the preference agreement and enjoyment relation. These results showed partial support for our third hypothesis.

### 4.1 Intensity selection/prescription as a relevant tool in a need‐supportive environment

On a first level of analysis, these results are promising for exploring how professionals could support BPN fulfillment in their regular work tasks with their clients, a topic that has dragged on in this field for a long time. Considering the amount and quality of evidence of the SDT framework in the exercise and sport and health‐related fields (e.g., Gillison et al., 2019; Ntoumanis et al., 2021; Ryan et al., 2022), it is appalling to see the absence of operational adjustments and applications resulting from it. As such, it is imperious to explore and develop interventions that could help the operationalization of the promised theoretical assumptions (Gillison et al., 2019; Rhodes et al., 2019). On a second level of analysis, it was possible to identify autonomy as the only psychological need that had an indirect association with the outcomes (i.e., enjoyment and frequency). As seen in previous studies in this context grounded on SDT, all BPN should, albeit more directly or indirectly through other motivational determinants, be related to the studied outcomes (e.g., Rodrigues et al., 2020; Rodrigues et al., 2023; Teixeira et al., 2018; Teixeira et al., 2021), something also detected in the studies that tested preference agreement and the psychological needs on these outcomes (Rodrigues et al., 2023; Teixeira, Rodrigues, et al. (2021)). Notwithstanding, the higher associations between preference agreement and autonomy, as for the detected indirect effects through this psychological need on the outcomes, tend to suggest that exercisers may be perceiving to have some choice and control in their session, even if it only derives from the selection of their exercise intensity. This is not unheard of, as some previous works that focused on understanding the relation between the use of intensity self‐selection versus imposed intensity have demonstrated (for a review, see Andrade et al., 2024).

Although results tend to show higher associations with autonomy, both competence and relatedness depicted positive associations with intensity preference. These differences may be related to the fact that it is easier for the exerciser to perceive that intensity is being adjusted because he wants (or has been told he can ask for it), thus favoring autonomy satisfaction perceptions, rather than for the adjustment of the degree of challenge (i.e., competence satisfaction) or empathy for the effort experienced (i.e., relatedness satisfaction). Notwithstanding, and although not reflecting more directly those potential influences on the studied outcome variables, one must consider that SDT presents them as crucial for the internalization and integration of the behavior, supporting the development of autonomous regulations, long presented as more adequate for the support of exercise adherence (Ryan & Deci, 2017; Ryan et al., 2021, 2022), and thus lending support for the relevance of this and future explorations regarding the use of intensity selection/prescription as a tool in a need‐supportive environment.

Finally, it is worth noting that there was a significant interaction between sex and the autonomy‐enjoyment path, suggestive of a moderating effect. In both cases, increases in the BPN of autonomy were positively associated with exercise enjoyment. Still, this effect was higher in males, a possible relevant consideration when aiming to understand enjoyment development between sexes. Previous efforts on the topic have already suggested that males were more prone to exercise due to exercise‐related enjoyment than females (Craft et al., 2014; Mao et al., 2020), but regarding SDT, no inherent difference is expected to exist in the way the BPN is associated with enjoyment. However, given that self‐determined motivation is a predictor of enjoyment (Murcia et al., 2008), future efforts should strive to understand if this difference between sexes is due to the BPN or autonomous regulation effects (or the specific contribution of each construct), the possible relation with intensity preference, and their possible difference among sexes. Enjoyment is a strong predictor of exercise adherence, and comprehending how to promote it is paramount for this endeavor.

### 4.2 Intensity preference agreement and exercise behavior: A distinct path targeting the same wanted outcome

In exercise settings, preference and tolerance have been considerably explored in the last few years (Santos & Teixeira, 2023). For example, Marques et al. (2022) showed in a retrospective study that intensity preference agreement was higher in exercisers enrolled in activities for longer than a year when compared with those training for less than a year, less than six months, and less than three months, proposing that one condition that could be sustaining long‐term behavior would be this agreement and how intensity was being experienced. Expanding on this idea, Teixeira et al. (2022) aimed to test the moderating effect of intensity preference agreement in the relationship between enjoyment and three relevant outcomes of exercise behavior (habit, intention, and exercise frequency). They have shown that preference agreement positively moderated those associations, suggesting that even the relation between a strong predictor of exercise adherence, as is enjoyment, could benefit from an individually adjusted exercise intensity. More recently, proposals for the use of preference agreement to prescribe exercise intensity targeting positive affective responses have emerged (Teixeira et al., 2023), adding to the literature now considering different pathways to promote and support motivational aspects in exercise practice. These are usually grounded on the idea that exercise behavior results from the interaction of reflective and automatic processing paths, which ultimately manifest in the realization (or not) of that behavior (Brand & Ekkekakis, 2018; Stevens et al., 2020). Additionally, the intensity pleasure–displeasure relation (i.e., promoting a positive affective regulation; ACSM, 2021) is presented in several theoretical frameworks as a downstream influence in both automatic and reflective paths, which may end, for example, in an exercise affective (positive or negative) association (automatic), the development of an affectively charged motivation (automatic‐reflective interaction), as a desire/dread for an activity or a context/activity specific intrinsic motivation, or the development (or not) of positive behavioral intentions (reflective) (Stevens et al., 2020; Williams, 2023). This is probably what justifies the positive associations between preference agreement and enjoyment and intention in the mediation models. As preference agreement increases, the exercise intensity may be experienced in a more individually preferred and pleasurable activity, thus reinforcing a set of influences deriving from a positive affective experience that would, automatically and reflectively, be related to improved enjoyment and intention.

As for the moderating effects detected, only positive effects were detected. Of those, two were related to females and one to males. Although still very exploratory at this point, it appears that higher scores in preference agreement are associated with higher levels of enjoyment, and this relation tends to increase more clearly in female exercisers. This may be unveiling that females may be more dependent than males on the pleasurable relation with exercise intensity. At the same time, men may be more dependent on autonomy for that purpose, a hypothesis supported by the conditional indirect effect analysis. As for the model where intention was tested as an outcome, the preference agreement association with this variable seems to be more strongly moderated in the male sample of exercisers. Although potentially relevant, the moderation analysis results may be dependent on several distinct factors like contextual characteristics (type of activity/session/class) (Rodrigues et al., 2023), fatigue threshold (Ansdell et al., 2020), physiological differences to exercise intensity exposure (Rascon et al., 2020), among other, which exploration is beyond the purpose of this study. Nonetheless, these constitute the first evidence of differences between sexes that could justify different approaches when developing individual exercise prescriptions based on intensity preference.

Potential suggestions arising from the current study echo recent research efforts on this topic, indicating that professionals should aim, whenever possible, to prescribe exercise intensity or advise on activities that align with the intensity individually preferred and that express pleasurable feelings. This could be obtained in a vast array of options, but for the particular case of supervised exercise settings, by manipulating training variables. Not only can the intensity magnitude be manipulated, but resting periods, cadence, frequency, and volume, to name some examples, can impact exercise dynamics and alter intensity perception at a given exercise or activity. For this, a pre‐evaluation of intensity preference (and eventually tolerance), as previously framed using an instrument for that purpose (e.g., PRETIE‐Q), as for the affective response assessment (for a broader look on this topic, see Williams & Rhodes, 2023), or simply through a clear and objective talk about the exerciser's experience during exercise, could achieve both an intensity preference agreement as for perceptions of a need‐supporting environment favorable of BPN development.

Independent of the path chosen (i.e., promoting the bright side of SDT or a hedonic approach to motivation development), several proxy variables of exercise frequency gain benefits worth contemplating when professionals consider exercise intensity for motivational support. In the struggle to help sustain exercise throughout the human lifespan, hardly one theory, method, strategy, or approach would suffice. Ensuring that professionals have easy‐to‐implement strategies grounded in strong theoretical foundations may be worth pursuing and should receive attention from researchers in the upcoming years.

### 4.3 Limitations and future studies

On a first note, despite being one of the first works to explore a SDT and a hedonic‐based approach to exercise behavior in exercise settings, much can be improved and better understood with a well‐balanced and experimental approach. The very nature of the mediation analysis process must be grounded on strong theoretical assumptions when applied to cross‐sectional studies (Hayes, 2018). As mentioned throughout our research, some theoretical connections are still absent of contextual and experimental evidence, limiting the depth of our potential analysis and cautioning when extrapolating the results for operational purposes.

On another note, it is important to consider that exercisers responded to a Likert scale question regarding the agreement with their current exercise intensity. Still, no exploration was made to understand if this agreement was made through the prescription of a professional, by a supervised self‐selection, or just by exercising on their own. For example, in a situation where non‐individualized supervision occurs, the exerciser may perceive a higher autonomy when contrasting with a more individualized process (e.g., personal training), and the contrary may be present in a group class. A more detailed exploration of the conditions and characteristics of the exercise intensity performed (e.g., intensity domain; affective response), the exerciser (e.g., experience), and the associated context/activities (e.g., type of activity; type of supervision) would be crucial for the deepening of these variables' relation understanding.

The exploration made in this study was grounded in two distinct but crucial rationales. First, exercise professionals could, while working with standard prescription variables (e.g., intensity), use their interventions to support BPN fulfillment, and second, an alignment of individual exercise intensity preference and current training intensity is conducive to a better affective response, which would lead to improved exercise enjoyment, intention, and frequency, thus adding cumulatively to the well‐known and established positive relation of BPN satisfaction and these outcomes. When thinking on the implementation side of our study results, we suggest considering the recommendations of the behavior change wheel (Michie et al., 2011), where our hypothesis can be further contextually explored in the framework related to the intervention function, targeting both the automatic and reflective aspects of motivation. Moreover, an SDT‐based motivationally adaptive communication style, as proposed by Ntoumanis et al. (2017) in a related context (i.e., group classes), may set the conditions not only to allow an intensity preference agreement but also for other autonomy‐supportive conditions conducive to improved enjoyment and exercise behavior. A deeper understanding of this can be seen in Ahmadi et al.’s (2023) classification system, where a comprehensive list of teacher's motivational behaviors consistent with SDT is presented.

In conclusion, this exploratory work presented preliminary support for the role of the agreement between the intensity individually preferred for a given activity and the one being realized in the current exercise session/training. This agreement depicted positive associations with exercise BPN fulfillment, enjoyment, and intention, and indirectly with exercise frequency through autonomy satisfaction. The present results could be considered an operational strategy to be used in a need‐supportive environment, targeting several exercise behavior variables relevant to exercise adherence, regardless of whether they are associated with automatic or reflective pathways of motivational development.

## CONFLICT OF INTEREST STATEMENT

No conflict of interest to declare.

## Supporting information

Supporting Information S1
