# Supplementary material for: Exercise intensity agreement, need satisfaction, and exercise behavior: A sex‐moderated mediation model
Source: Eur J Sport Sci. 2024 Sep 16;24(10):1495–507. doi: 10.1002/ejsc.12173 (PMC11451574; doi:10.1002/ejsc.12173)
Supplement: Supplementary file 1 — Supporting Information S1 [file EJSC-24-1495-s001.docx]

Supplementary File 1 - Descriptive and group comparison analysis by sex. *Note.* M = mean; SD = standard deviation; t = t-test; *p* = significance

|  | Male | | Female | |  |  |
| --- | --- | --- | --- | --- | --- | --- |
|  | M | SD | M | SD | t | *p* |
| Preference Agreement | 4.42 | 0.79 | 4.46 | 0.79 | .52 | .60 |
| Autonomy Satisfaction | 4.28 | 0.63 | 4.27 | 0.61 | -.25 | .80 |
| Competence Satisfaction | 4.46 | 0.55 | 4.39 | 0.58 | -1.21 | .23 |
| Relatedness Satisfaction | 4.15 | 0.78 | 4.25 | 0.82 | 1.07 | .29 |
| Autonomy Frustration | 1.83 | 0.87 | 1.68 | 0.74 | -1.73 | .08 |
| Competence Frustration | 1.66 | 0.70 | 1.79 | 0.78 | 1.60 | .11 |
| Relatedness Frustration | 1.50 | 0.62 | 1.39 | 0.57 | -1.86 | .06 |
| Enjoyment | 5.98 | 0.97 | 6.18 | 0.88 | 2.14 | .04 |
| Intention to continue | 6.30 | 1.01 | 6.47 | 0.92 | 1.63 | .10 |
| Exercise Frequency | 3.97 | 1.22 | 3.95 | 1.19 | -.14 | .89 |
